# Rice *OseIF6.1* encodes a eukaryotic translation initiation factor and is essential for the development of grain and anther

**DOI:** 10.3389/fpls.2024.1366986

**Published:** 2024-03-21

**Authors:** Hongming Guo, Jianqun Lv, Xiangwen Su, Liang Chen, Juansheng Ren, Liping Liu, Mingxin Ren, Song Liu, Mingli Dai, Guangjun Ren, Fangyuan Gao

**Affiliations:** ^1^ Environment-Friendly Crop Germplasm Innovation and Genetic Improvement Key Laboratory of Sichuan Province, Crop Research Institute, Sichuan Academy of Agricultural Sciences, Chengdu, China; ^2^ Key Laboratory of Tianfu Seed Industry Innovation (Co-construction by Ministry and Province), Ministry of Agriculture and Rural Affairs, Chengdu, China; ^3^ Xiamen Key Laboratory for Plant Genetics, School of Life Sciences, Xiamen University, Xiamen, China

**Keywords:** OseIF6.1, eukaryotic translation initiation factor, grain shape, pollen sterility, seed setting

## Abstract

The eIF6 proteins are distributed extensively in eukaryotes and play diverse and essential roles. The bona fide eIF6 protein in *Arabidopsis*, At-eIF6;1, is essential for embryogenesis. However, the role of eIF6 proteins in rice growth and development remains elusive and requires further investigation. Here, we characterized the functions of OseIF6.1, which is homologous to At-eIF6;1. *OseIF6.1* encodes an eukaryotic translation initiation factor with a conserved eIF6 domain. The knockdown of *OseIF6.1* resulted in a decrease in grain length and pollen sterility, whereas the overexpression of *OseIF6.1* displayed opposite phenotypes. Further studies revealed that *OseIF6.1* regulates grain shape by influencing cell expansion and proliferation. In addition, OseIF6.1 interacts with OsNMD3, which is a nuclear export adaptor for the 60S ribosomal subunit. The knockdown of *OsNMD3* in plants exhibited reduced fertility and seed setting. Therefore, our findings have significantly enriched the current understanding of the role of *OseIF6.1* in rice growth and development.

## 1 Introduction

Protein synthesis is a complex process that can be categorized into four distinct stages: initiation, elongation, termination, and ribosome recycling (Sonenberg and Hinnebusch, 2009). Translation initiation represents the crucial phase of protein synthesis, where the ribosome assembles on the mRNA and initiates the synthesis of the polypeptide chain (Merrick and Pavitt, 2018). In eukaryotes, this process is regulated and carried out by a series of protein complexes known as eukaryotic translation initiation factors (eIFs) (Gebauer and Hentze, 2004; Hinnebusch, 2006). These factors orchestrate the assembly of the translation initiation complex, which includes the small ribosomal subunit, initiator tRNA, and mRNA (Merrick and Pavitt, 2018). The eIFs not only facilitate the correct positioning of the ribosome on the mRNA but also regulate the rate and efficiency of translation initiation (Roy and von Arnim, 2013; Raabe et al., 2019). As such, they serve as important regulators of gene expression and can influence various aspects of plant growth and development.

The types of eIFs are diverse and complex, and at least 29 have been identified, including eIF1, eIF1A, eIF2, eIF2α, eIF2B, eIF3a-m, eIF4A1, eIF4A2, eIF4B, eIF4E, eIF4G, eIF4F, eIF5, eIF5A, eIF5B, eIF6.1and eIF6.2. Each exerting distinct roles during the translation initiation process (Jackson et al., 2010; Guo et al., 2011; Aitken and Lorsch, 2012; Hinnebusch and Lorsch, 2012; Raabe et al., 2019; Castellano and Merchante, 2021; Singha et al., 2021; Ma et al., 2022). Among them, eIF6 is an essential protein that possesses a distinctive anti-association activity. As it binds to immature large ribosomal subunits (pre-60S) in the nucleolus, it prevents their premature association with 40S subunits (Miluzio et al., 2009). Subsequently, eIF6 is detected in the nucleoplasm during pre-60S subunit maturation and is exported to the cytosol where it releases the 60S ribosomal subunit. The 60S subunits then join with 40S subunits to form the 80S ribosome complex (Si and Maitra, 1999; Basu et al., 2001; Ceci et al., 2003; Gandin et al., 2008; Miluzio et al., 2009).

Over the past decades, extensive research has been devoted to exploring the function of eIFs in animals and yeast (Kapp and Lorsch, 2004; Sonenberg and Hinnebusch, 2009; Aitken and Lorsch, 2012). Indeed, recent research has suggested that eIFs play a crucial role in regulating cell differentiation, cell cycle progression, and stress responses in plants (Rausell et al., 2003; Thompson et al., 2004; Diédhiou et al., 2008; Singh et al., 2013; Chen et al., 2019; Raabe et al., 2019; Castellano and Merchante, 2021). For example, *Fumonisin B1-resistant 12* (*FBR12*) encodes a putative eIF-5A-2 protein that regulates growth and development of floral organs and sporogenesis by influencing cell division, cell growth, and cell death in *Arabidopsis* (Feng et al., 2007). In addition, *eIF5A* played a significant role in the process of cadmium (Cd) accumulation and sensitivity in *Arabidopsis*. The *ateif5a* mutant exhibited a higher level of Cd accumulation in both roots and shoots compared to the wild type. Moreover, *AteIF5A* was found to impact Cd sensitivity by modulating Cd uptake, accumulation, and detoxification (Xu et al., 2015). Furthermore, eIFs are involved in the processes of plant growth and development, such as embryogenesis, flowering, and organogenesis. For example, mutations in the *eIF3* subunits *eIF3e*, *eIF3f*, and *eIF3h* in *Arabidopsis* do not impact pollen formation or maturation. However, they do cause deficiencies in pollen germination and/or pollen tube growth, resulting in reduced efficiency of male gamete transmission (Xia et al., 2010; Roy et al., 2011). Rice eIF3 subunit f has been reported to play an important role in post-meiotic pollen formation, knockdown of *OseIF3f* showed a large reduction in seed setting and pollen fertility (Li et al., 2016). The expression levels of *OseIF3f* were significantly higher in unicellular microspores and bicellular pollen than in mature tricellular pollen or germinated pollen, with at least a three-fold increase observed, suggesting that *OseIF3f* might play a more crucial role in microgametogenesis rather than pollen germination (Wei et al., 2010). Interestingly, OseIF3e interacted with OseIF3f and OseIF6, respectively. *OseIF3e*-RNAi plants exhibited stunted growth during both the seedling and vegetative stages, and displayed defects in pollen maturation and small grains (Wang et al., 2016).

eIF6 was first discovered and characterized as a wheat protein that associates with the 60S ribosome in crop plants (Russell and Spremulli, 1980). The study on eIF6 in yeast (TIF6) demonstrated its crucial role in ribosome biogenesis. When TIF6 is depleted, it causes abnormal processing of ribosomal RNA (rRNA) precursors and a decrease in the abundance of 60S ribosomal subunits, leading to a lethal phenotype (Wood et al., 1999; Basu et al., 2001). Human eIF6 (p27BBP) interacts with 60S ribosome subunits, preventing the assembly of both 40S and 60S subunits in the cytosol (Sanvito et al., 1999). Embryos lacking *eIF6* in mice exhibit a lethal phenotype during the preimplantation stage. Additionally, heterozygous mice display insensitivity to insulin and show reduced hepatic and adipose tissue mass, as well as a decrease in protein synthesis (Gandin et al., 2008). The *Arabidopsis at-eif6;1* mutant exhibits an embryonic-lethal phenotype, similar to yeast and mouse. One-third of the pale yellow seeds were observed in heterozygotes mutant siliques (Kato et al., 2010). However, little is known about the function of *eIF6* in crop plants, despite these studies indicating a critical role for *eIF6* in embryogenesis.

Our previous study has shown that OseIF6.1 interacts with OsLa, and it was impossible to isolate a homozygous *oseif6.1* mutant, as the plants displayed abnormal floral organs, leading to a lethal phenotype (Guo et al., 2022). However, the function of *OseIF6.1* gene has yet to be characterized. Our present study aimed to validate the roles of *OseIF6.1* in the growth and development of rice. In this study, we identified and characterized the functions of *OseIF6.1* by using RNA interference (RNAi) approach. We found that *OseIF6.1* encodes a protein homologous to At-eIF6;1 in *Arabidopsis*, and *OseIF6.1* acts as a positive regulator in grain size and pollen sterility. In addition, OseIF6.1 physically interacts with OsNMD3, which is a nuclear export adaptor for the 60S ribosomal subunit. The *OsNMD3*-RNAi lines exhibited a phenotype of reduced fertility. Therefore, these findings help to reveal the function of *OseIF6.1* in rice grain size and pollen fertility.

## 2 Materials and methods

### 2.1 Plant materials and growth conditions

The *japonica* rice (*Oryza sativa*) cultivar (Nipponbare, Nip) was used for genetic transformation. Seeds of the wild type (WT), as well as the *OseIF6.1* and *OsNMD3* transgenic lines, underwent sterilization using a 10% sodium hypochlorite (NaClO) solution for a duration of 30 minutes. After this, they were rinsed thoroughly five times with sterile water. The sterilized seeds were planted in sterile plastic containers half-strength Murashige and Skoog (MS) medium. These containers were subsequently placed in a controlled growth chamber, maintaining a photoperiod of 16 hours of light at an approximate temperature of 28 ± 2°C, followed by 8 hours of darkness at around 25 ± 2°C. The chamber’s relative humidity was kept between 70-85%. After a two-week period of incubation, the seedlings were carefully transplanted to field conditions. All plants were planted in the experimental field of Crop Research Institute, Sichuan Academy of Agricultural Sciences, Chengdu, China. Each plant was separated by 20 cm within each row, and the rows were also spaced 20 cm apart. The field management was essentially based on standard agricultural practices.

### 2.2 Phylogenetic and conserved domains analysis

The OseIF6.1 protein homolog sequences were obtained from the National Center for Biotechnology Information (NCBI) database (http://www.ncbi.nlm.nih.gov/). These sequences were aligned to construct phylogenetic trees based on the maximum-likelihood (ML) criterion, employing 1000 bootstraps in MEGA 5. The prediction of their conserved domains was carried out using the NCBI Batch CD-search tool (https://www.ncbi.nlm.nih.gov/Structure/bwrpsb/bwrpsb.cgi) and TBtools (Chen et al., 2020).

### 2.3 Plasmid construction and rice transformation

For the construction of the *OseIF6.1* overexpression vector, the complete coding sequence of *OseIF6.1* was PCR-amplified from Nipponbare cDNA, followed by its subsequent cloning into the pCXUN-Flag vector using TA cloning method (Chen et al., 2009). For the construction of the *OseIF6.1* and *OsNMD3* Knockdown vector, the gene-specific sequences of *OseIF6.1* and *OsNMD3* were cloned into the pH7GWIWGII with the LR Clonase II enzyme (Invitrogen), respectively.

We introduced recombinant plasmids into Nipponbare callus tissues of rice using the *Agrobacterium tumefaciens* strain EHA105, employing the previously described transformation protocol (Hiei et al., 1994). All the primers used are listed in **Supplementary Table 1**.

### 2.4 Localization of OseIF6.1

To determine the subcellular localization of OseIF6.1 protein, its CDS was inserted into the pCXDG vector, creating a fusion with green fluorescent protein (GFP), which was driven by the CaMV35 promoter. The vector was introduced into the *A. tumefaciens* strain GV3101, followed by the transient transformation into the leaves of *Nicotiana benthamiana* at the age of four weeks. Confocal microscopy (LSM 780, Carl Zeiss) was used to observe GFP fluorescence signals.

### 2.5 RNA isolation and quantitative real-time PCR

Young panicles and seedlings of WT, and *OseIF6.1* transgenic lines were used to isolate total RNA using an Vazyme FastPure Universal Plant Total RNA Isolation Kit. First-strand cDNA was synthesized using Vazyme’s HiScript II Q RT SuperMix for qPCR with gDNA wiper. Quantitative real-time PCR (qRT-PCR) was conducted using a QuantStudio Flex PCR system machine (Thermo Fisher Scientific) with Powerup™ SYBR™ Green Master Mix (Applied Biosystems). The Actin gene of Nip was chosen as the internal reference, and the relative expression levels of the target genes were quantified employing the 2^-ΔΔCt^ method (Livak and Schmittgen, 2001). All the primers used are listed in **Supplementary Table 1**.

### 2.6 Phenotype analysis

WT and transgenic plants were grown in a rice field and photographed at maturity. Grain traits including plant height, primary branch, secondary branch, panicle length, grain length, grain width, grain thickness, and 1000-grain weight, were measured. Mature grains were dried and sprayed with gold, and then observed using a scanning electron microscope. Cell number and size were determined using Image J software. The sample preparation and TEM observation were carried out according to the previous method for semi-thin section and ultra-thin section assays (Cao et al., 2018).

### 2.7 Yeast two-hybrid assays

The *OseIF6.1* and *OsNMD3* CDS have been cloned into plasmids pGBKT7 and pGADT7, respectively. Ligation-independent cloning (LIC) was used to construct all plasmids (Aslanidis et al., 1994). The Yeast two-hybrid (Y2H) assay was conducted in accordance with the manufacturer’s instructions (Clontech). The pGBKT7-53 plasmid was transformed with pGADT7-T as a positive control, and the pGBKT7-Lam plasmid was transformed with pGADT7-T into the Y2HGold strain as a negative control. All the primers used are listed in **Supplementary Table 1**.

### 2.8 Bimolecular fluorescence complementation assay

The CDS of *OseIF6.1* was cloned into the N-terminal fragment of the p2YN vector, and the CDS of *OsNMD3* was cloned into the C-terminal fragment of the p2YC vector. The LIC method was used to construct all plasmids. For transient expression, *N. benthamiana* leaves were coinfiltrated with *A. tumefaciens* strain GV3101 carrying different plasmid combinations and the p19 strain. The confocal microscopy (LSM 780, Carl Zeiss) was used to observe the fluorescent signals of the yellow fluorescent protein (YFP). All the primers used are listed in **Supplementary Table S1**.

### 2.9 Pull-down assay

The CDSs of *OseIF6.1* and *OsNMD3* were cloned into pET-28a (+) and pET-GST vectors, respectively, to generate His-OseIF6.1 and GST-OsNMD3 proteins. All plasmids were constructed using the LIC method, and subsequently transformed into the *Escherichia coli* BL21 strain. A pull down assay was performed as previously described (Guo et al., 2022). The precipitates were analyzed by western blot using GST and His antibodies (Thermo Fisher Scientific). All the primers used are listed in **Supplementary Table 1**.

### 2.10 Statistical analysis

Data are means ± SE from at least three independent experiments. The Statistical analysis was carried out using IBM SPSS software (IBM Corp., Armonk, NY, USA), and means were compared by Student’s t-tests.

## 3 Results

### 3.1 OseIF6.1 is conserved in eukaryotes

**Figure 1:**
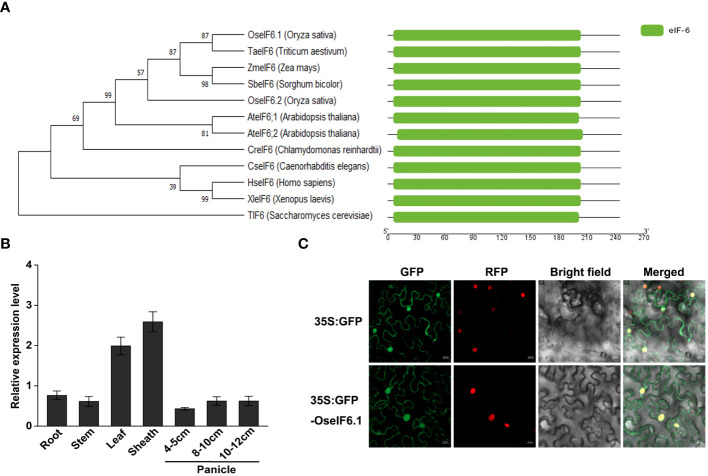
Expression pattern and subcellular localization of OseIF6.1. **(A)** Comparative analysis of eIF6 protein phylogeny and structure across diverse eukaryotic species. **(B)** Relative expression of *OseIF6.1* in root, stem, leaf, and sheath of young seedlings and developing panicles of 4-5, 8-10, and 10-12 cm. *OsActin* served as an internal control. The values represent means ± SE derived from at least three independent experiments. **(C)** Subcellular localization of OseIF6.1 in *N. benthamiana* leaves. Scale bars = 20 μm.

First, the eIF6 homologs in eukaryotes were identified by running a BLAST search against the *Arabidopsis* At-eIF6;1 protein in the NCBI database. A multiple protein sequence alignment of eIF6 protein homologs from *Oryza sativa* (Genbank accession XP_015647337.1; OseIF6.1), *Oryza sativa* (XP_015635348.1; OseIF6.2), *Arabidopsis thaliana* (NP_191121.1; At-eIF6;1), *Arabidopsis thaliana* (NP_181512.1; At-eIF6;2), *Homo sapiens* (CAX12724.1; HseIF6), and *Saccharomyces cerevisiae* (NP_015341.1; TIF6) revealed that the sequences of the six proteins are highly similar (**Supplementary Figure 1**). An analysis of phylogenetic relationships and structural comparisons between eIF6 proteins from various eukaryotes revealed that they contain a conserved eIF6 domain, as well as OseIF6.1 shares a close relationship with other homologues, and that eIF6 protein is conserved across eukaryotes (**Figure 1A**).

### 3.2 Expression pattern and subcellular localization of OseIF6.1

In order to elucidate the expression profile of *OseIF6.1* in rice tissues, we employed qRT-PCR assay. The expression levels of OseIF6.1 transcripts were examined in the root, stem, leaf, and sheath tissues of young seedlings, as well as in developing panicles at three different stages: 4-5 cm, 8-10 cm, and 10-12 cm. qRT-PCR analysis revealed that the expression of *OseIF6.1* was detected in all tested tissues, with particularly high expression levels observed in leaves and sheaths (**Figure 1B**). To investigate the subcellular localization of OseIF6.1, a GFP-OseIF6.1 fusion protein driven by the CaMV 35S promoter was transiently expressed in *N. benthamiana* leaves. Similar to the GFP signal, confocal images showed that GFP-OseIF6.1 was detected in the cytoplasm, and nucleus (**Figure 1C**).

### 3.3 Ectopic *OseIF6.1* expression affects the morphology of plant and grain

**Figure 2:**
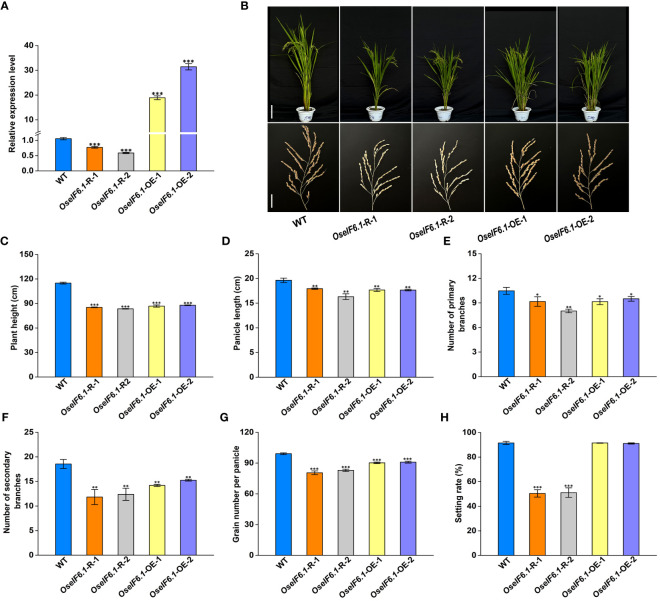
Phenotypic analysis of *OseIF6.1*-overexpressing and knockdown in rice. **(A)** Relative expression levels of OseIF6.1 in WT and OseIF6.1 transgenic lines. *OsActin* served as an internal control. The values represent means ± SE derived from at least three independent experiments. **(B)** Plants and Panicles of WT and *OseIF6.1* transgenic plants at the mature stage. Scale bars = 20 cm for plant height and 3 cm for panicle length. **(C)** Plant heights of WT and *OseIF6.1* transgenic plants. **(D)** Panicle length of WT and *OseIF6.1* transgenic plants. **(E)** Number of primary branches of WT and *OseIF6.1* transgenic panicles. **(F)** Number of secondary branches of WT and *OseIF6.1* transgenic panicles. **(G)** Grain number per panicle of WT and *OseIF6.1* transgenic plants. **(H)** Setting rate of WT and *OseIF6.1* transgenic plants. The values represent means ± SE derived from at least three independent experiments. Student’s *t*-test: *p < 0.05, **p < 0.01, ***p < 0.001.

To investigate the function of *OseIF6.1*, we generated *OseIF6.1* knockdown transgenic lines by RNA interference (RNAi) technology. Additionally, OseIF6.1 was tagged with a Flag under the control of a maize ubiquitin promoter to obtain overexpression lines. These vectors were successfully introduced into Nipponbare through an *Agrobacterium tumefaciens*-mediated transformation. Further functional analysis was conducted on two OE and two knockdown lines (**Figure 2A**). The relative expression levels of *OseIF6.1* in *OseIF6.1*-R-1 and *OseIF6.1*-R-2 were reduced by 0.77-fold and 0.59-fold, respectively, compared to that in WT. In contrast, the expression levels of *OseIF6.1* in *OseIF6.1*-OE-1 and *OseIF6.1*-OE-2 were 18.9-fold and 31.4-fold higher, respectively, than that of WT (**Figure 2A**).

At the mature stage, several agronomic traits between WT and *OseIF6.1* transgenic lines were measured (**Figure 2B**). As shown in **Figures 2C, D**, the *OseIF6.1*-R and *OseIF6.1*-OE plants had reduced plant height and shorter panicles than the WT. In addition, we found that the *OseIF6.1*-R and *OseIF6.1*-OE plants had fewer primary and secondary branches, as well as grain number per panicle compared with the WT (**Figures 2E–G**). Interestingly, the setting rate in *OseIF6.1*-R plants was dramatically lower when compared with the WT and *OseIF6.1*-OE lines, while there was no significant difference observed between the WT and *OseIF6.1*-OE lines (**Figure 2H**).

**Figure 3:**
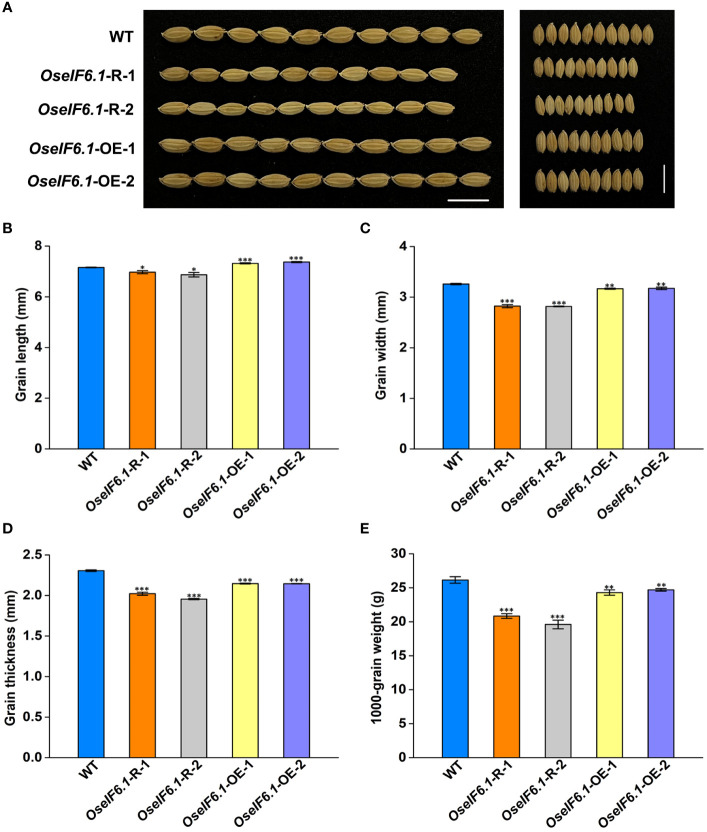
Ectopic expression of *OseIF6.1* alters the grain shape. **(A)** Mature paddy grains of WT and *OseIF6.1* transgenic lines. Scale bars =1 cm. **(B)** Grain length of WT and *OseIF6.1* transgenic lines. **(C)** Grain width of WT and *OseIF6.1* transgenic lines. **(D)** Grain thickness of WT and *OseIF6.1* transgenic lines. **(E)** 1000-grain weight of WT and *OseIF6.1* transgenic lines. Student’s *t*-test: *p < 0.05, **p < 0.01, ***p < 0.001.

Further analysis suggested that *OseIF6.1* acts as a positive regulator of grain length. When compared with WT, the *OseIF6.1*-R plants exhibited shorter and smaller grains (**Figures 3A–C**). However, the grain lengths in the *OseIF6.1*-OE1 and OE2 lines were significantly increased by 2.24% and 2.97%, respectively, than those of the WT (**Figures 3A, B**). Furthermore, both *OseIF6.1*-R and *OseIF6.1*-OE grains exhibited a decrease in grain width and thickness compared to WT grains (**Figures 3C, D**), leading to their lower 1000-grain weights compared to WT (**Figure 3E**). Therefore, the findings suggest that OsEIF6.1 plays a pivotal role in rice growth and development.

### 3.4 *OseIF6.1* influences grain size by regulating cell expansion and proliferation

**Figure 4:**
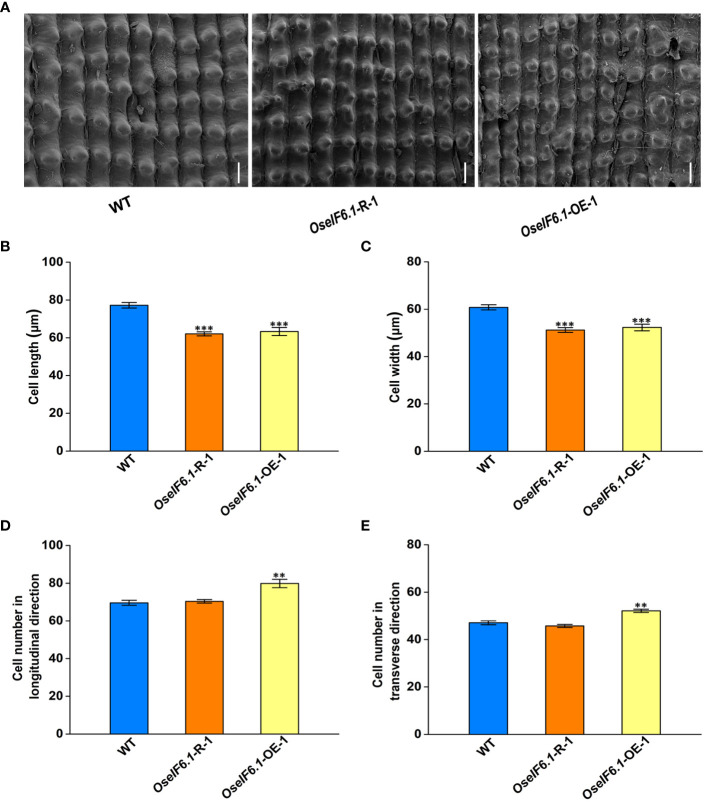
OseIF6.1 regulates grain size by affecting cell expansion and cell proliferation. **(A)** Scanning electron microscopy images of the glume outer surfaces of WT and OseIF6.1 transgenic lines mature grains. Scale bars = 50 μm. Average length **(B)** and width **(C)** of the outer epidermal cells of WT and OseIF6.1 transgenic lines lemmas. **(D)** Outer epidermal cell number in the longitudinal direction of WT and OseIF6.1 transgenic lines lemmas. **(E)** Outer epidermal cell number in the transverse direction of WT and OseIF6.1 transgenic lines lemmas. The values represent means ± SE derived from at least three independent experiments. Student’s *t*-test: *p < 0.05, **p < 0.01, ***p < 0.001.

The spikelet hull size is a crucial factor in promoting grain size growth, as it relies on coordinated cell proliferation and expansion (Li and Li, 2016). To comprehensively assess the impact of OseIF6.1 on grain size, we utilized scanning electron microscopy (SEM) to meticulously examine the outer glume of both WT and OseIF6.1 transgenic plants’ spikelet hulls (**Figure 4A**). The outer epidermal cells in OseIF6.1-R and OseIF6.1-OE lemma were shorter and narrower than those of the WT (**Figures 4B, C**). In contrast, the number of cells in the outer glume of OseIF6.1-OE was significantly higher than in WT in both the longitudinal and transverse directions (**Figures 4D, E**). On the other hand, the cell numbers in OseIF6.1-R spikelet hulls were lower than those of the WT (**Figures 4D, E**). Together, these results suggest that OseIF6.1 plays a positive role in regulating grain size by influencing cell expansion and cell proliferation.

### 3.5 Knockdown of *OseIF6.1* affects pollen fertility

Previous results have shown that knockdown of OseIF6.1 leads to a reduced seed setting rates. To further investigate this finding, we conducted a pollen viability assay using KI-I_2_ staining on both WT and OseIF6.1 transgenic plants (**Supplementary Figure 2A**). The KI-I_2_ staining rates were 95.7%, 95.6%, and 96.1% in WT and two OseIF6.1-OE anthers, respectively, while the corresponding values were only 48.6% and 50.3% in OseIF6.1-R-1 and OseIF6.1-R-2 anthers, respectively (**Supplementary Figure 2B**). The lower KI-I_2_ staining rates in OseIF6.1-R anthers suggest a possible role for OseIF6.1 in the regulation of meiotic progression.

**Figure 5:**
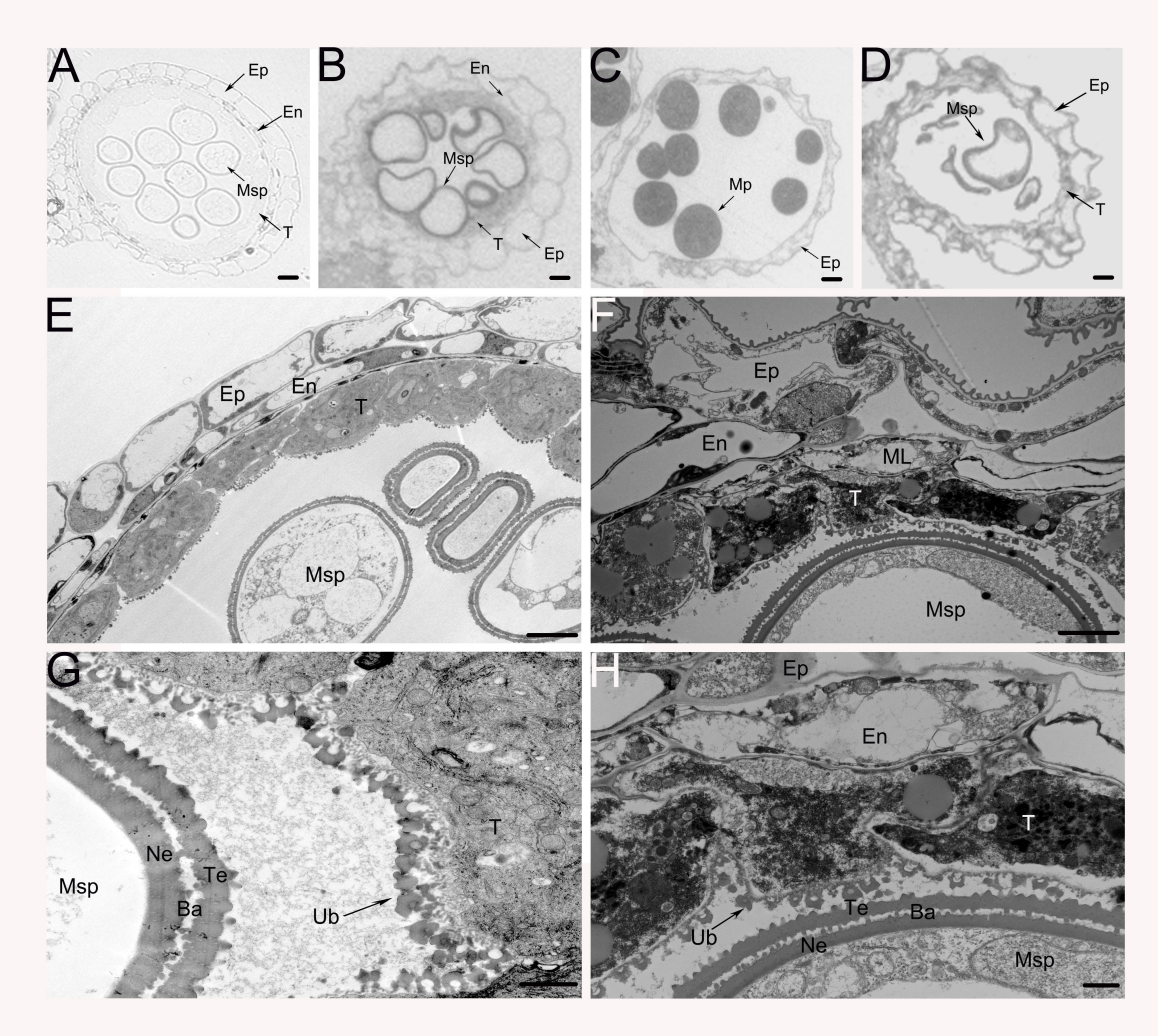
Transverse section and comparison between WT and OseIF6.1 knockdown transgenic anthers. Cross sections of WT **(A, C)** and OseIF6.1 knockdown transgenic lines **(B, D)** anthers at stage 10 **(A, B)** and stage 12 **(C, D)**. Scale bars = 50 μm. Transmission electron microscopy images of WT **(E, G)** and OseIF6.1 knockdown transgenic lines **(F, H)** anthers at stage 10. Scale bars = 5 μm in **(E)** and **(F)**. Scale bars = 1 μm in **(G)** and **(H)**. Ba, bacula; En, endothecium; Ep, epidermis; ML, middle layer; Msp, microspores; MP, mature pollen; Ne, nexine; T, tapetum; Te, tectum; Ub, Ubisch body.

To gain a deeper understanding of the cytological defects in OseIF6.1-R anthers, we conducted a comprehensive analysis of WT and OseIF6.1-R anthers at various stages of development through semithin section assays. During Stage 10, the WT tapetum continues to degrade, with the entire layer of tapetal cells present as a band-like structure. The middle layer is almost completely degraded, and the spherical microspores show multiple small vacuoles (**Figure 5A**). In contrast, the OseIF6.1-R plants exhibited irregular microspore morphology, with an abnormal distribution of the tapetal cell layer (**Figure 5B**). Only epidermal cells remained in the WT anthers, the tapetum layer had completely degraded, and the microspores had eventually developed into mature pollen at stage 12 (**Figure 5C**). However, the OseIF6.1-R anthers exhibit incomplete degradation of the tapetum layer, along with degenerated microspores that show a sickle shape and lack starch accumulation (**Figure 5D**). To further investigate the defects in anther development of OseIF6.1-R, the transmission electron microscope (TEM) was used to perform ultrathin section assays. As shown in **Figures 5E, G**, the WT tapetum layer is filled with numerous small vacuoles and organelles, and the surface of the tapetum layer cells is abundant in Ubisch bodies, which play an important role in the transport of substances between the tapetum layer and the microspores. However, the *OseIF6.1*-R tapetal layer contains fewer intracellular contents and has a reduced number of Ubisch bodies on its surface (**Figures 5F, H**). Thus, the results obtained in this study demonstrate that the observed pollen defects in *OseIF6.1-R* anthers are likely linked to tapetal degradation.

To test this hypothesis, we conducted an analysis of the expression of genes related to anther development in both WT and *OseIF6.1*-R young panicles. The expression of *PTC2*, *TDR*, and *EAT1*, which play a crucial role in tapetum programmed cell death (PCD) and pollen wall formation (Zhang et al., 2008; Niu et al., 2013; Uzair et al., 2020), was found to be downregulated in *OseIF6.1-R* plants compared to WT (**Supplementary Figure 3**). In addition, similar expression tendency of *DPW* and *CYP703A3* was also observed in *OseIF6.1*-R plants (**Supplementary Figure 3**), which regulates sporopollenin precursor biosynthesis (Shi et al., 2011; Yang et al., 2014). Taken together, these findings suggest that *OseIF6.1* positively regulates seed setting and pollen viability, and may also affect anther development by altering the expression of related genes.

### 3.6 OseIF6.1 physically interacts with OsNMD3

**Figure 6:**
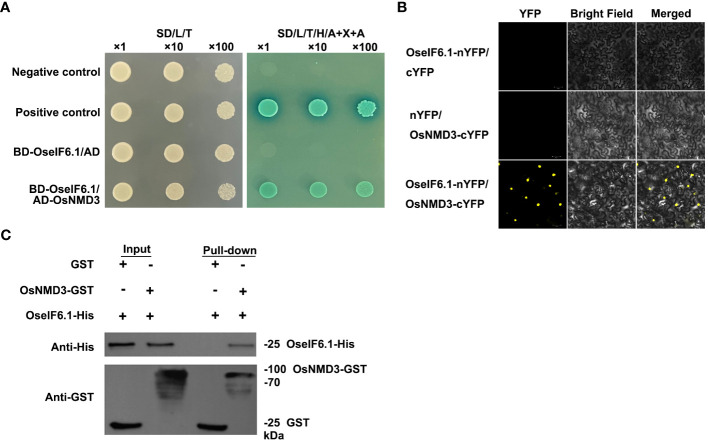
OseIF6.1 physically interacts with OsNMD3. **(A)** OseIF6.1 interacts with OsNMD3 in yeast cells. Transformed cells were cultured on DDO or QDO/X/A media. **(B)** Bimolecular fluorescence complementation assays verifies the interaction between OseIF6.1 and OsNMD3 in *N. benthamiana*. OseIF6.1-nYFP was coexpressed with OsNMD3-cYFP in cells of *N. benthamiana*. Scale bars = 50 μm. **(C)** Pull-down assay indicates that OseIF6.1 binds OsNMD3 *in vitro*. OseIF6.1-His was incubated with OsNMD3-GST and pulled down by OsNMD3-GST.

To further investigate the potential function of OseIF6.1 in rice growth and development, we utilized the publicly accessible rice interactome network to identify proteins that interact with OseIF6.1 (https://bar.utoronto.ca/eplant_rice/ and http://bioinfo.sibs.ac.cn/plant-regulomics/index.php/ Waese et al., 2017; Ran et al., 2020). In the list of proteins predicted to interact with OseIF6.1, OsNMD3 was identified as a potential candidate, which encodes a nuclear export adaptor for the 60S ribosomal subunit. As expected, the yeast two-hybrid (Y2H) results showed that OseIF6.1 interacted with OsNMD3 (**Figure 6A**). The interaction between OseIF6.1 and OsNMD3 was further confirmed by bimolecular fluorescence complementation (BiFC) assays. OseIF6.1 and OsNMD3 were fused to the N-terminal and C-terminal of YFP, respectively. By co-expressing the OseIF6.1-nYFP and OsNMD3-cYFP constructs in *N. benthamiana* cells, the reconstitution of YFP fluorescence indicated the direct binding of these two proteins *in vivo* (**Figure 6B**). Furthermore, we conducted a pull-down assay to investigate whether OseIF6.1 can engage in direct interaction with OsNMD3 *in vitro*. We expressed and purified His-tagged OseIF6.1 (OseIF6.1-His) and glutathione S-transferase (GST) tag-fused OsNMD3 (OsNMD3-GST) in *Escherichia coli*, respectively. As shown in **Figure 6C**, OseIF6.1-His bound to OsNMD3-GST, but not to the negative control. Thus, these findings suggest that OseIF6.1 can interact with OsNMD3.

### 3.7 Knockdown of OsNMD3 alters plant architecture and pollen fertility

**Figure 7:**
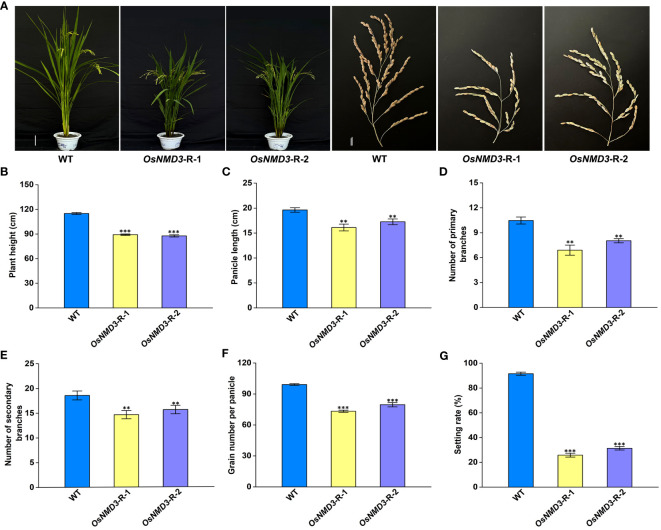
Knockdown of OsNMD3 affects multiple agronomic traits. **(A)** Plants and Panicles of WT and OsNMD3 knockdown transgenic plants at the mature stage. Scale bars = 20 cm for plant height and 3 cm for panicle length. **(B)** Plant heights of WT and OsNMD3 knockdown transgenic plants. **(C)** Panicle length of WT and OsNMD3 knockdown transgenic plants. **(D)** Number of primary branches of WT and OsNMD3 knockdown transgenic panicles. **(E)** Number of secondary branches of WT and OsNMD3 knockdown transgenic panicles. **(F)** Grain number per panicle of WT and OsNMD3 knockdown transgenic plants. **(G)** Setting rate of WT and OsNMD3 knockdown transgenic plants. The values represent means ± SE derived from at least three independent experiments. Student’s *t*-test: *p < 0.05, **p < 0.01, ***p < 0.001.

We employed RNAi approach to generate *OsNMD3* knockdown transgenic lines, as we were concerned that gene editing of *OsNMD3* may result in a lethal phenotype similar to that of the *oseif6.1* mutant. Two *OsNMD3*-R lines were chosen for further analysis. The expression levels of *OsNMD3* were reduced by 0.52 and 0.69 fold in *OsNMD3*-R-1 and *OsNMD3*-R-2, respectively, compared to that in WT (**Supplementary Figure 4**). Subsequently, we conducted an assessment of agronomic traits in mature WT and *OsNMD3-R* plants (**Figure 7A**). The *OsNMD3-R* plants exhibited shorter plant height and smaller panicles compared to the WT (**Figures 7B, C**). Furthermore, the *OsNMD3*-R plants had fewer primary and secondary branches than the WT, resulting in a decrease in grain number per panicle (**Figures 7D–F**). Interestingly, the setting rate in OsNMD3-R plants was significantly lower compared to the WT (**Figure 7G**). We then performed pollen viability assays on WT and OsNMD3-R anthers using KI-I_2_ staining. The results showed that the pollen viability of OsNMD3-R-1 and OsNMD3-R-2 anthers was 31.8% and 34.2%, respectively, which was markedly lower compared to the WT anthers (**Supplementary Figure 5**). Taken together, these results suggest that the knockdown of OsNMD3 has negative impacts on plant architecture and pollen viability.

## 4 Discussion

Originally purified from wheat germ, eukaryotic initiation factor 6 (eIF6) was discovered to function as a ribosome dissociation factor (Russell and Spremulli, 1980). By binding to the 60S ribosome subunit, eIF6 prevents its association with the 40S ribosome subunit (Russell and Spremulli, 1980). Homologous proteins of wheat eIF6 were subsequently isolated and purified from various sources, including rabbit (Raychaudhuri et al., 1984), calf, human (Si et al., 1997), yeast (Si and Maitra, 1999), and *Arabidopsis* (Kato et al., 2010). The phylogenetic analysis indicates that the eIF6 protein is conserved among eukaryotes (**Figure 1A**). Structural comparisons of eIF6 proteins have further revealed that OseIF6.1 shares a close relationship with other homologues (**Supplementary Figure 1**). Several studies on yeast and mammals have demonstrated the crucial role of eIF6 in growth regulation. Depletion of eIF6 is lethal in both yeast and mouse models (Si et al., 1997; Sanvito et al., 1999; Wood et al., 1999; Gandin et al., 2008). Like in yeast and mouse, the null allele of the *At-eIF6;1* in *Arabidopsis* results in an embryonic-lethal phenotype, underscoring the essential role of *eIF6* in plant embryogenesis (Kato et al., 2010). Although numerous studies have examined the role of eIF6 proteins in eukaryotes, our previous study demonstrated that *oseif6.1* mutant was unable to harvest seeds, which resulted in a lethal phenotype (Guo et al., 2022). Therefore, the functional characterization of the *OseIF6.1* gene remains to be fully understood.

Given that the mutant of *OseIF6.1* results in embryonic lethality, we conducted an investigation into the function of *OseIF6.1* in rice by either knocking down or overexpressing its expression in Nipponbare. In this study, we found that *OseIF6.1* plays an important role in the development of plant architecture and grain shape. Both the knockdown and overexpression of *OseIF6.1* resulted in a dwarfing phenotype (**Figure 2A**). It has been widely reported that gibberellins (GA) are the primary regulators of plant height, influencing the growth and development of stems and determining the overall stature of the plant (Salas Fernandez et al., 2009). The expression of the genes related to GA biosynthesis in stems of WT and OseIF6.1 transgenic plants was examined. We found that the expression of *OsGA20ox1* (Oikawa et al., 2004), *OsGA20ox2* (Sasaki et al., 2002; Su et al., 2021), and *OsKO2* (Itoh et al., 2004) was suppressed in *OseIF6.1*-R and *OseIF6.1*-OE stems (**Supplementary Figure 6**). These results suggest that altered *eIF6*.1 expression affects the expression of genes related to GA biosynthesis and ultimately plant height. Furthermore, the knockdown of *OseIF6.1* resulted in short, narrow, and thin grains, whereas the overexpression of *OseIF6.1* resulted in long grains (**Figures 3A–D**), indicating that *OseIF6.1* acts as a positive regulator of grain shape. The conclusion was reinforced through scanning electron microscopy observations, which disclosed that *OseIF6.1* has a positive impact on grain size by regulating cell elongation and proliferation (**Figure 4**). Therefore, *OseIF6.1* seems to be a key determinant of plant structure and rice grain morphology. These lines exhibit no difference in seed germination compared to the WT. However, the *OseIF6.1*-R and OE lines both exhibit a reduced 1000-grain weight compared to the WT (**Figure 3E**), potentially due to changes in *OseIF6.1* expression that affect the translation efficiency of mRNAs related to grain development. Despite the *OseIF6.1*-OE lines having increased translation efficiency, there is a decrease in both grain width and thickness. This could potentially be attributed to compensatory mechanisms or feedback regulation, resulting in a similar 1000-grain weight phenotype to that observed in the *OseIF6.1*-R lines.

The *At-eIF6;1* heterozygous plants exhibit a 1:3 ratio of growth defects in their silique seeds, which display a pale yellow coloration (Kato et al., 2010). Interestingly, we have noted that plants with *OseIF6.1-R* show decreased seed-setting rates alongside diminished pollen fertility (**Figure 2H**; **Supplementary Figure 2**). Subsequent analysis using transmission electron microscopy revealed that the abortion of *OseIF6.1*-R pollens is associated with the degradation of the tapetum layer (**Figure 5**). Numerous investigations have identified a range of genes that play a pivotal role in tapetum PCD and the formation of pollen walls. These genes include *Udt1* (Jung et al., 2005), *Wda1* (Jung et al., 2006), *TDR* (Zhang et al., 2008), *DPW* (Shi et al., 2011), *EAT1* (Niu et al., 2013), *CYP703A3* (Yang et al., 2014), and *PTC2* (Uzair et al., 2020). We observed that the expression of *CYP703A3*, *PTC2*, *TDR*, *EAT1* and *DPW* genes was downregulated in *OseIF6.1*-R young panicles (**Supplementary Figure 3**). These findings suggest that *OseIF6.1* is essential for anther development. Consistent with this, the depletion of *eIF6* in yeast causes a reduction in the availability of free 60S ribosomal subunits, resulting in a decrease in translational activity, which is eventually lethal (Wood et al., 1999; Basu et al., 2001). In addition, it has been reported that *eIF6*-null embryos are lethal at the preimplantation stage in mice, and the quality of hepatic and adipose tissue in the heterozygous *eIF6* mice is reduced due to the reduction in cell number and perturbation of the G1/S cell cycle process (Gandin et al., 2008). Together with our results, these data suggest that *OseIF6.1* plays a vital role in embryonic development across eukaryotes.

The biogenesis of ribosomes is a complex process that involves various trans-acting factors. eIF6 can bind to immature large ribosomal subunits as well as other trans-acting factors in the nucleolus (Miluzio et al., 2009). *NMD3* is a conserved transcriptional factor that encodes the nuclear export adaptor for the 60S ribosomal subunit. This protein is characterized by its N-terminus, which contains Cx2C repeats and a nuclear localization sequence. The C-terminus of NMD3 features a nuclear export sequence, which is essential for maintaining the efficiency of normal protein synthesis (Shi et al., 2014). Overexpression of *OsNMD3^ΔNLS^*, which lacks a nuclear localization site, caused abnormal plant growth and development in rice, including dwarfism and reduced grain size (Shi et al., 2014). In the *Arabidopsis AtNMD3ΔNES* OE line, pleiotropic phenotypes were observed, such as reduced plant height and stamen size, as well as an obvious curly shape of the rosette leaves (Chen et al., 2012). NMD3 may interact with specific ribosomal proteins, especially those associated with the 60S ribosomal subunit, facilitating their proper assembly and export from the nucleus to the cytoplasm, given the roles of its homologs in other species (Hedges et al., 2006; Sengupta et al., 2010; Chen et al., 2012). It has been reported that OsNMD3 interacts with the 60S subunit through OsRPL10Ac1 (Shi et al., 2014). In this study, we have discovered that OseIF6.1 interacts with OsNMD3 (**Figure 6**). Interestingly, the *OsNMD3*-R plants exhibit similar phenotypic traits to those of *OseIF6.1*-R plants. The *OsNMD3*-R plants also exhibit reduced plant height and pollen fertility (**Figure 7**; **Supplementary Figure 5**). The expression of the GA biosynthesis-related genes, including *OsGA20ox1*, *OsGA20ox2*, and *OsKO2* were decreased in stems of *OsNMD3*-R plants (**Supplementary Figure 7**). Similar dwarfism and sterility phenomena have also been observed in other *eIFs* RNAi plants. For example, it has been reported that OseIF3e protein interacts with OseIF6. Notably, RNAi targeting *OseIF3e* causes significant plant height and pollen maturation defects (Wang et al., 2016). The mutation of *OseIF3h* has been found to cause abnormal development in rice, particularly affecting plant height and pollen fertility (Huang et al., 2021). Similarly, the T-DNA mutants of *AteIF3e* exhibited diminished pollen fertility, along with various floral and reproductive defects. Overexpressing *AteIF3e* led to dwarfism and affected seed formation (Yahalom et al., 2008). The differences in seed setting between the *OseIF6.1*-R and *OsNMD3*-R lines suggest that they may regulate rice growth via distinct or partially overlapping pathways. Higher seed setting rates in *OseIF6.1*-R lines imply that its role might be partly independent of *OsNMD3*, indicating the existence of additional pathways or partners for *OseIF6.1*. The more significant effect observed with *OsNMD3* reduction could indicate that *OsNMD3* operates downstream of *OseIF6.1* or in a parallel pathway that is crucial for rice fertility. In future studies, genetic epistasis analysis can elucidate their relationships in rice development.

In conclusion, our findings indicate that *OseIF6.1* plays a regulatory role in the expression of sporopollenin precursor biosynthesis genes, as well as genes involved in tapetal PCD, thereby influencing tapetal development and the formation of pollen. Additionally, it modulates the expression of GA biosynthesis-related genes, thus regulating plant height. Furthermore, OseIF6.1 interacts with OsNMD3, which also plays a crucial role in regulating pollen fertility and plant height. By identifying and characterizing OseIF6.1, the research provides insights into the genetic factors that influence rice growth and development. Understanding these genetic components is crucial for breeding and genetic engineering efforts aimed at crop improvement. The specific roles of OseIF6.1 in regulating grain length and fertility highlight it as a potential target for genetic engineering.

## Data availability statement

The datasets presented in this study can be found in online repositories. The names of the repository/repositories and accession number(s) can be found in the article/**Supplementary Material**.

## Author contributions

HG: Investigation, Writing – original draft, Writing – review & editing, Data curation. JL: Data curation, Writing – original draft. XS: Investigation, Writing – original draft. LC: Data curation, Investigation, Writing – original draft. JR: Data curation, Writing – original draft. LL: Data curation, Writing – original draft. MR: Writing – original draft. SL: Writing – original draft. MD: Writing – original draft. GR: Writing – original draft, Writing – review & editing. FG: Writing – original draft, Writing – review & editing.

## Supplementary material

Supplementary Figure 1 Alignment of eIF6 homologues from *Oryza sativa*, *Arabidopsis thaliana*, *Homo sapiens*, and *Saccharomyces cerevisiae*.

Supplementary Figure 2 Fertility analysis of WT and *OseIF6.1* transgenic plants. **(A)** KI-I_2_ staining of WT, *OseIF6.1*-R-1, and *OseIF6.1*-OE-1 pollen. Normal pollen appears dark colored. Scale bars = 500 μm. **(B)** KI-I_2_ staining rate of WT and *OseIF6.1* transgenic plants pollen. The values represent means ± SE derived from at least three independent experiments. Student’s *t*-test: *p < 0.05, **p < 0.01, ***p < 0.001.

Supplementary Figure 3 Expression levels of genes involved in anther development in WT and *OseIF6.1* knockdown transgenic plants young panicles. The values represent means ± SE derived from at least three independent experiments. Student’s *t*-test: *p < 0.05, **p < 0.01, ***p < 0.001.

Supplementary Figure 4 OsNMD3 expression in WT and OsNMD3 knockdown transgenic lines. The values represent means ± SE derived from at least three independent experiments. Student’s *t*-test: *p < 0.05, **p < 0.01, ***p < 0.001.

Supplementary Figure 5 Fertility analysis of WT and OsNMD3 knockdown transgenic plants. **(A)** KI-I_2_ staining of WT and OsNMD3 knockdown transgenic lines pollen. Normal pollen appears dark colored. Scale bars = 1 mm. **(B)** KI-I_2_ staining rate of WT and OsNMD3 knockdown transgenic lines pollen. The values represent means ± SE derived from at least three independent experiments. Student’s *t*-test: *p < 0.05, **p < 0.01, ***p < 0.001.

Supplementary Figure 6 Expression levels of genes involved in gibberellin biosynthesis in stems of WT and *OseIF6.1* transgenic plants. The values represent means ± SE derived from at least three independent experiments. Student’s *t*-test: *p < 0.05, **p < 0.01, ***p < 0.001.

Supplementary Figure 7 Expression levels of genes involved in gibberellin biosynthesis in stems of WT and *OsNMD3* knockdown transgenic plants. The values represent means ± SE derived from at least three independent experiments. Student’s *t*-test: *p < 0.05, **p < 0.01, ***p < 0.001.

Supplementary Figure 8 A working model for the role of OseIF6.1 in rice growth and development.

## References

[B1] Aitken C. E. Lorsch J. R. (2012). A mechanistic overview of translation initiation in eukaryotes. Nat. Struct. Mol. Biol. 19, 568–576. doi: 10.1038/nsmb.2303 22664984

[B2] Aslanidis C. de Jong P. J. Schmitz G. (1994). Minimal length requirement of the single-stranded tails for ligation-independent cloning (LIC) of PCR products. PCR Methods Appl. 4, 172–177. doi: 10.1101/gr.4.3.172 7580902

[B3] Basu U. Si K. Warner J. R. Maitra U. (2001). The *Saccharomyces cerevisiae TIF6* gene encoding translation initiation factor 6 is required for 60S ribosomal subunit biogenesis. Mol. Cell Biol. 21, 1453–1462. doi: 10.1128/MCB.21.5.1453-1462.2001 11238882 PMC86691

[B4] Cao J. B. Cheng K. Yuan M. (2018). Observation of rice tissues with semi-thin section. Bio 101, e1010142. doi: 10.21769/BioProtoc.1010142

[B5] Castellano M. M. Merchante C. (2021). Peculiarities of the regulation of translation initiation in plants. Curr. Opin. Plant Biol. 63, 102073. doi: 10.1016/j.pbi.2021.102073 34186463

[B6] Ceci M. Gaviraghi C. Gorrini C. Sala L. A. Offenhäuser N. Marchisio P. C.. (2003). Release of eIF6 (p27^BBP^) from the 60S subunit allows 80S ribosome assembly. Nature 426, 579–584. doi: 10.1038/nature02160 14654845

[B7] Chen C. Chen H. Zhang Y. Thomas H. R. Frank M. H. He Y.. (2020). TBtools: an integrative toolkit developed for interactive analyses of big biological data. Mol. Plant 13, 1194–1202. doi: 10.1016/j.molp.2020.06.009 32585190

[B8] Chen K. Guo T. Li X. M. Zhang Y. M. Yang Y. B. Ye W. W.. (2019). Translational regulation of plant response to high temperature by a dual-function tRNA^His^ guanylyltransferase in rice. Mol. Plant 12, 1123–1142. doi: 10.1016/j.molp.2019.04.012 31075443

[B9] Chen M. Q. Zhang A. H. Zhang Q. Zhang B. C. Nan J. Li X.. (2012). *Arabidopsis* NMD3 is required for nuclear export of 60S ribosomal subunits and affects secondary cell wall thickening. PloS One 7, e35904. doi: 10.1371/journal.pone.0035904 22558264 PMC3338764

[B10] Chen S. Songkumarn P. Liu J. Wang G. L. (2009). A versatile zero background T-vector system for gene cloning and functional genomics. Plant Physiol. 150, 1111–1121. doi: 10.1104/pp.109.137125 19403729 PMC2705043

[B11] Diédhiou C. J. Popova O. V. Dietz K. J. Golldack D. (2008). The SUI-homologous translation initiation factor *eIF-1* is involved in regulation of ion homeostasis in rice. Plant Biol. 10, 298–309. doi: 10.1111/j.1438-8677.2008.00037.x 18426477

[B12] Feng H. Chen Q. Feng J. Zhang J. Yang X. Zuo J. (2007). Functional characterization of the Arabidopsis eukaryotic translation initiation factor 5A-2 that plays a crucial role in plant growth and development by regulating cell division, cell growth, and cell death. Plant Physiol. 144, 1531–1545. doi: 10.1104/pp.107.098079 17513484 PMC1914145

[B13] Gandin V. Miluzio A. Barbieri A. M. Beugnet A. Kiyokawa H. Marchisio P. C.. (2008). Eukaryotic initiation factor 6 is rate-limiting in translation, growth and transformation. Nature 455, 684–688. doi: 10.1038/nature07267 18784653 PMC2753212

[B14] Gebauer F. Hentze M. W. (2004). Molecular mechanisms of translational control. Nat. Rev. Mol. Cell Biol. 5, 827–835. doi: 10.1038/nrm1488 15459663 PMC7097087

[B15] Guo H. Cui Y. Huang L. Ge L. Xu X. Xue D.. (2022). The RNA binding protein OsLa influences grain and anther development in rice. Plant J. 110, 1397–1414. doi: 10.1111/tpj.15746 35322500

[B16] Guo J. Jin Z. Yang X. Li J. Chen J. (2011). Eukaryotic initiation factor 6, an evolutionarily conserved regulator of ribosome biogenesis and protein translation. Plant Signal Behav. 6, 766–771. doi: 10.4161/psb.6.5.15438 21543889 PMC3172860

[B17] Hedges J. Chen Y. West M. Bussiere C. Johnson A. W. (2006). Mapping the functional domains of yeast NMD3, the nuclear export adapter for the 60 S ribosomal subunit. J. Biol. Chem. 281, 36579–36587. doi: 10.1074/jbc.M606798200 17015443

[B18] Hiei Y. Ohta S. Komari T. Kumashiro T. (1994). Efficient transformation of rice (*Oryza sativa* L.) mediated by Agrobacterium and sequence analysis of the boundaries of the T-DNA. Plant J. 6, 271–282. doi: 10.1046/j.1365-313X.1994.6020271.x 7920717

[B19] Hinnebusch A. G. (2006). eIF3: a versatile scaffold for translation initiation complexes. Trends Biochem. Sci. 31, 553–562. doi: 10.1016/j.tibs.2006.08.005 16920360

[B20] Hinnebusch A. G. Lorsch J. R. (2012). The mechanism of eukaryotic translation initiation: new insights and challenges. Cold Spring Harb. Perspect. Biol. 4, a011544. doi: 10.1101/cshperspect.a011544 22815232 PMC3475172

[B21] Huang Y. Zheng P. Liu X. Chen H. Tu J. (2021). OseIF3h Regulates Plant Growth and Pollen Development at Translational Level Presumably through Interaction with OsMTA2. Plants (Basel). 10, 1101. doi: 10.3390/plants10061101 34070794 PMC8228589

[B22] Itoh H. Tatsumi T. Sakamoto T. Otomo K. Toyomasu T. Kitano H.. (2004). A rice semi-dwarf gene, *Tan-Ginbozu* (*D35*), encodes the gibberellin biosynthesis enzyme, ent-kaurene oxidase. Plant Mol. Biol. 54, 533–547. doi: 10.1023/B:PLAN.0000038261.21060.47 15316288

[B23] Jackson R. J. Hellen C. U. Pestova T. V. (2010). The mechanism of eukaryotic translation initiation and principles of its regulation. Nat. Rev. Mol. Cell Biol. 11, 113–127. doi: 10.1038/nrm2838 20094052 PMC4461372

[B24] Jung K. H. Han M. J. Lee Y. S. Kim Y. W. Hwang I. Kim M. J.. (2005). Rice *Undeveloped Tapetum1* is a major regulator of early tapetum development. Plant Cell. 17, 2705–2722. doi: 10.1105/tpc.105.034090 16141453 PMC1242267

[B25] Jung K. H. Han M. J. Lee D. Y. Lee Y. S. Schreiber L. Franke R.. (2006). *Wax-deficient anther1* is involved in cuticle and wax production in rice anther walls and is required for pollen development. Plant Cell. 18, 3015–3032. doi: 10.1105/tpc.106.042044 17138699 PMC1693940

[B26] Kapp L. D. Lorsch J. R. (2004). The molecular mechanics of eukaryotic translation. Annu. Rev. Biochem. 73, 657–704. doi: 10.1146/annurev.biochem.73.030403.080419 15189156

[B27] Kato Y. Konishi M. Shigyo M. Yoneyama T. Yanagisawa S. (2010). Characterization of plant eukaryotic translation initiation factor 6 (eIF6) genes: the essential role in embryogenesis and their differential expression in *Arabidopsis* and rice. Biochem. Biophys. Res. Commun. 397, 673–678. doi: 10.1016/j.bbrc.2010.06.001 20570652

[B28] Li Q. Deng Z. Gong C. Wang T. (2016). The rice eukaryotic translation initiation factor 3 subunit f (OseIF3f) is involved in microgametogenesis. Front. Plant Sci. 7. doi: 10.3389/fpls.2016.00532 PMC4844609 27200010

[B29] Li N. Li Y. (2016). Signaling pathways of seed size control in plants. Curr. Opin. Plant Biol. 33, 23–32. doi: 10.1016/j.pbi.2016.05.008 27294659

[B30] Livak K. J. Schmittgen T. D. (2001). Analysis of relative gene expression data using real-time quantitative PCR and the 2^-ΔΔ C^ _T_ Method. Methods 25, 402–408. doi: 10.1006/meth.2001.1262 11846609

[B31] Ma L. Yang Y. Wang Y. Cheng K. Zhou X. Li J.. (2022). SlRBP1 promotes translational efficiency *via* SleIF4A2 to maintain chloroplast function in tomato. Plant Cell. 34, 2747–2764. doi: 10.1093/plcell/koac104 35385118 PMC9252502

[B32] Merrick W. C. Pavitt G. D. (2018). Protein synthesis initiation in eukaryotic cells. Cold Spring Harb. Perspect. Biol. 3, a033092. doi: 10.1101/cshperspect.a033092 PMC6280705 29735639

[B33] Miluzio A. Beugnet A. Volta V. Biffo S. (2009). Eukaryotic initiation factor 6 mediates a continuum between 60S ribosome biogenesis and translation. EMBO Rep. 10, 459–465. doi: 10.1038/embor.2009.70 19373251 PMC2680881

[B34] Niu N. Liang W. Yang X. Jin W. Wilson Z. A. Hu J.. (2013). EAT1 promotes tapetal cell death by regulating aspartic proteases during male reproductive development in rice. Nat. Commun. 4, 1445. doi: 10.1038/ncomms2396 23385589

[B35] Oikawa T. Koshioka M. Kojima K. Yoshida H. Kawata M. (2004). A role of *OsGA20ox1*, encoding an isoform of gibberellin 20-oxidase, for regulation of plant stature in rice. Plant Mol. Biol. 55, 687–700. doi: 10.1007/s11103-004-1692-y 15604710

[B36] Raabe K. Honys D. Michailidis C. (2019). The role of eukaryotic initiation factor 3 in plant translation regulation. Plant Physiol. Biochem. 145, 75–83. doi: 10.1016/j.plaphy.2019.10.015 31665669

[B37] Ran X. Zhao F. Wang Y. Liu J. Zhuang Y. Ye L.. (2020). Plant Regulomics: a data-driven interface for retrieving upstream regulators from plant multi-omics data. Plant J. 101, 237–248. doi: 10.1111/tpj.14526 31494994

[B38] Rausell A. Kanhonou R. Yenush L. Serrano R. Ros R. (2003). The translation initiation factor eIF1A is an important determinant in the tolerance to NaCl stress in yeast and plants. Plant J. 34, 257–267. doi: 10.1046/j.1365-313X.2003.01719.x 12713533

[B39] Raychaudhuri P. Stringer E. A. Valenzuela D. M. Maitra U. (1984). Ribosomal subunit antiassociation activity in rabbit reticulocyte lysates. Evidence for a low molecular weight ribosomal subunit antiassociation protein factor (Mr = 25,000). J. Biol. Chem. 259, 11930–11935. doi: 10.1016/S0021-9258(20)71300-1 6566675

[B40] Roy B. Copenhaver G. P. Von Arnim A. G. (2011). Fluorescence-tagged transgenic lines reveal genetic defects in pollen growth-application to the eIF3 complex. PloS One 6, e17640. doi: 10.1371/journal.pone.0017640 21408229 PMC3049774

[B41] Roy B. von Arnim A. G. (2013). Translational regulation of cytoplasmic mRNAs. Arabidopsis Book. 11, e0165. doi: 10.1199/tab.0165 23908601 PMC3727577

[B42] Russell D. W. Spremulli L. L. (1980). Mechanism of action of the wheat germ ribosome dissociation factor: interaction with the 60 S subunit. Arch. Biochem. Biophys. 201, 518–526. doi: 10.1016/0003-9861(80)90540-8 6901609

[B43] Salas Fernandez M. G. Becraft P. W. Yin Y. Lübberstedt T. (2009). From dwarves to giants? plant height manipulation for biomass yield. Trends Plant Sci. 14, 454–461. doi: 10.1016/j.tplants.2009.06.005 19616467

[B44] Sanvito F. Piatti S. Villa A. Bossi M. Lucchini G. Marchisio P. C.. (1999). The β4 integrin interactor p27^BBP/eIF6^ is an essential nuclear matrix protein involved in 60S ribosomal subunit assembly. J. Cell Biol. 144, 823–837. doi: 10.1083/jcb.144.5.823 10085284 PMC2148184

[B45] Sasaki A. Ashikari M. Ueguchi-Tanaka M. Itoh H. Nishimura A. Swapan D.. (2002). Green revolution: a mutant gibberellin-synthesis gene in rice. Nature 416, 701–702. doi: 10.1038/416701a 11961544

[B46] Sengupta J. Bussiere C. Pallesen J. West M. Johnson A. Frank J. (2010). Characterization of the nuclear export adaptor protein Nmd3 in association with the 60S ribosomal subunit. J. Cell Biol. 189, 1079–1086. doi: 10.1083/jcb.201001124 20584915 PMC2894450

[B47] Shi Y. Liu X. Li R. Gao Y. Xu Z. Zhang B.. (2014). Retention of OsNMD3 in the cytoplasm disturbs protein synthesis efficiency and affects plant development in rice. J. Exp. Bot. 65, 3055–3069. doi: 10.1093/jxb/eru150 24723395 PMC4071826

[B48] Shi J. Tan H. Yu X. H. Liu Y. Liang W. Ranathunge K.. (2011). *Defective pollen wall* is required for anther and microspore development in rice and encodes a fatty acyl carrier protein reductase. Plant Cell. 23, 2225–2246. doi: 10.1105/tpc.111.087528 21705642 PMC3160036

[B49] Si K. Chaudhuri J. Chevesich J. Maitra U. (1997). Molecular cloning and functional expression of a human cDNA encoding translation initiation factor 6. Proc. Natl. Acad. Sci. U.S.A. 194, 14285–14290. doi: 10.1073/pnas.94.26.14285 PMC24943 9405604

[B50] Si K. Maitra U. (1999). The *Saccharomyces cerevisiae* homologue of mammalian translation initiation factor 6 does not function as a translation initiation factor. Mol. Cell Biol. 19, 1416–1426. doi: 10.1128/MCB.19.2.1416 9891075 PMC116070

[B51] Singh B. Chauhan H. Khurana J. P. Khurana P. Singh P. (2013). Evidence for the role of wheat eukaryotic translation initiation factor 3 subunit g (*TaeIF3g*) in abiotic stress tolerance. Gene 532, 177–185. doi: 10.1016/j.gene.2013.09.078 24084365

[B52] Singha D. L. Maharana J. Panda D. Dehury B. Modi M. K. Singh S. (2021). Understanding the thermal response of rice eukaryotic transcription factor *eIF4A1* towards dynamic temperature stress: insights from expression profiling and molecular dynamics simulation. J. Biomol Struct. Dyn. 39, 2575–2584. doi: 10.1080/07391102.2020.1751295 32367760

[B53] Sonenberg N. Hinnebusch A. G. (2009). Regulation of translation initiation in eukaryotes: mechanisms and biological targets. Cell 136, 731–745. doi: 10.1016/j.cell.2009.01.042 19239892 PMC3610329

[B54] Su S. Hong J. Chen X. Zhang C. Chen M. Luo Z.. (2021). Gibberellins orchestrate panicle architecture mediated by DELLA-KNOX signalling in rice. Plant Biotechnol. J. 19, 2304–2318. doi: 10.1111/pbi.13661 34245650 PMC8541776

[B55] Thompson J. E. Hopkins M. T. Taylor C. Wang T. W. (2004). Regulation of senescence by eukaryotic translation initiation factor 5A: implications for plant growth and development. Trends Plant Sci. 9, 174–179. doi: 10.1016/j.tplants.2004.02.008 15063867

[B56] Uzair M. Xu D. Schreiber L. Shi J. Liang W. Jung K. H.. (2020). PERSISTENT TAPETAL CELL2 is required for normal tapetal programmed cell death and pollen wall patterning. Plant Physiol. 182, 962–976. doi: 10.1104/pp.19.00688 31772077 PMC6997677

[B57] Waese J. Fan J. Pasha A. Yu H. Fucile G. Shi R.. (2017). ePlant: visualizing and exploring multiple levels of data for hypothesis generation in plant biology. Plant Cell. 29, 1806–1821. doi: 10.1105/tpc.17.00073 28808136 PMC5590499

[B58] Wang W. Xu M. Liu X. Tu J. (2016). The rice eukaryotic translation initiation factor 3 subunit e (OseIF3e) influences organ size and pollen maturation. Front. Plant Sci. 7. doi: 10.3389/fpls.2016.01399 PMC5028392 27703462

[B59] Wei L. Q. Xu W. Y. Deng Z. Y. Su Z. Xue Y. Wang T. (2010). Genome-scale analysis and comparison of gene expression profiles in developing and germinated pollen in *Oryza sativa* . BMC Genomics 11, 338. doi: 10.1186/1471-2164-11-338 20507633 PMC2895629

[B60] Wood L. C. Ashby M. N. Grunfeld C. Feingold K. R. (1999). Cloning of murine translation initiation factor 6 and functional analysis of the homologous sequence *YPR016c* in *Saccharomyces cerevisiae* . J. Biol. Chem. 274, 11653–11659. doi: 10.1074/jbc.274.17.11653 10206977

[B61] Xia C. Wang Y. J. Li W. Q. Chen Y. R. Deng Y. Zhang X. Q.. (2010). The Arabidopsis eukaryotic translation initiation factor 3, subunit F (*AteIF3f*), is required for pollen germination and embryogenesis. Plant J. 63, 189–202. doi: 10.1111/j.1365-313X.2010.04237.x 20444226 PMC7190160

[B62] Xu X. Y. Ding Z. J. Chen L. Yan J. Y. Li G. X. Zheng S. J. (2015). An eukaryotic translation initiation factor, *AteIF5A-2*, affects cadmium accumulation and sensitivity in *Arabidopsis* . J. Integr. Plant Biol. 57, 848–858. doi: 10.1111/jipb.12329 25559189

[B63] Yahalom A. Kim T. H. Roy B. Singer R. von Arnim A. G. Chamovitz D. A. (2008). Arabidopsis eIF3e is regulated by the COP9 signalosome and has an impact on development and protein translation. Plant J. 53, 300–311. doi: 10.1111/j.1365-313X.2007.03347.x 18067529

[B64] Yang X. Wu D. Shi J. He Y. Pinot F. Grausem B.. (2014). Rice CYP703A3, a cytochrome P450 hydroxylase, is essential for development of anther cuticle and pollen exine. J. Integr. Plant Biol. 56, 979–994. doi: 10.1111/jipb.12212 24798002

[B65] Zhang D. S. Liang W. Q. Yuan Z. Li N. Shi J. Wang J.. (2008). Tapetum degeneration retardation is critical for aliphatic metabolism and gene regulation during rice pollen development. Mol. Plant 1, 599–610. doi: 10.1093/mp/ssn028 19825565

