# Supplementary figures and images for: Rice OseIF6.1 encodes a eukaryotic translation initiation factor and is essential for the development of grain and anther
Source: Front Plant Sci. 2024 Mar 21;15:1366986. doi: 10.3389/fpls.2024.1366986 (PMC10991840; doi:10.3389/fpls.2024.1366986)

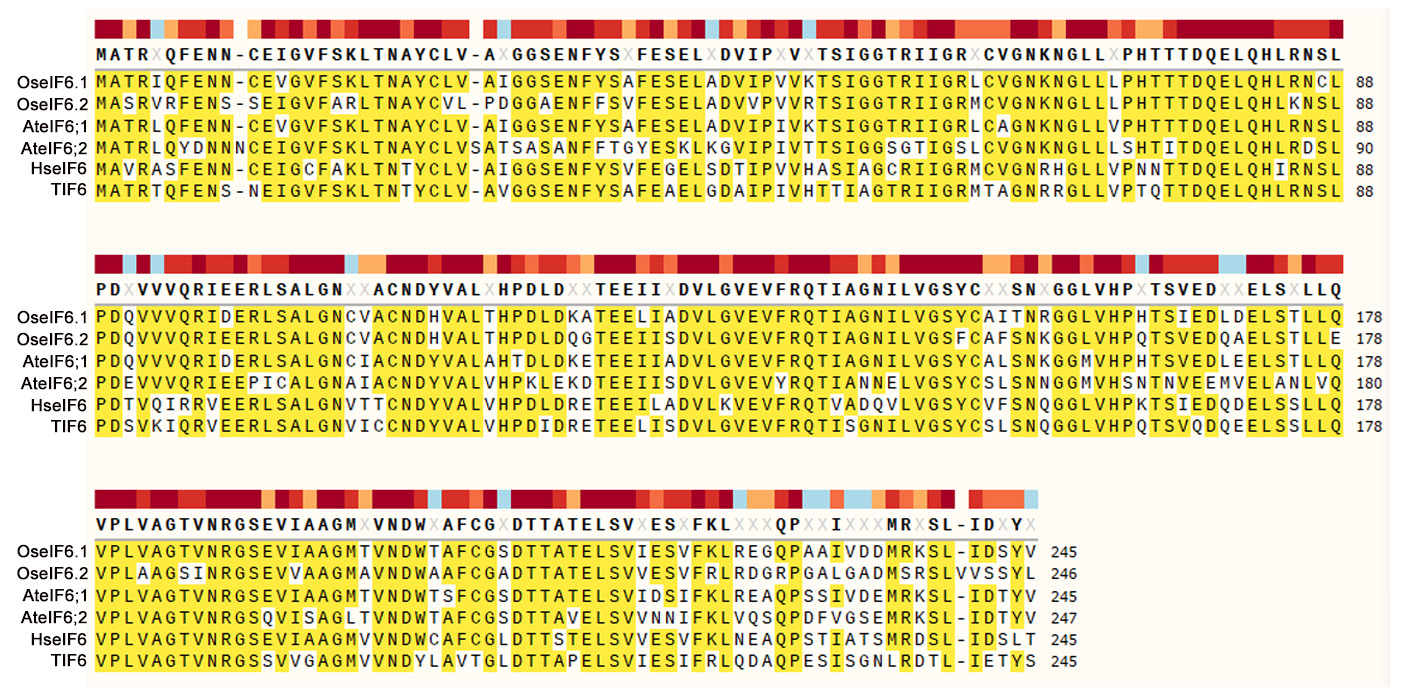

Supplement: Supplementary Figure 1 — Alignment of eIF6 homologues from Oryza sativa, Arabidopsis thaliana, Homo sapiens, and Saccharomyces cerevisiae. [file Image_1.jpeg]

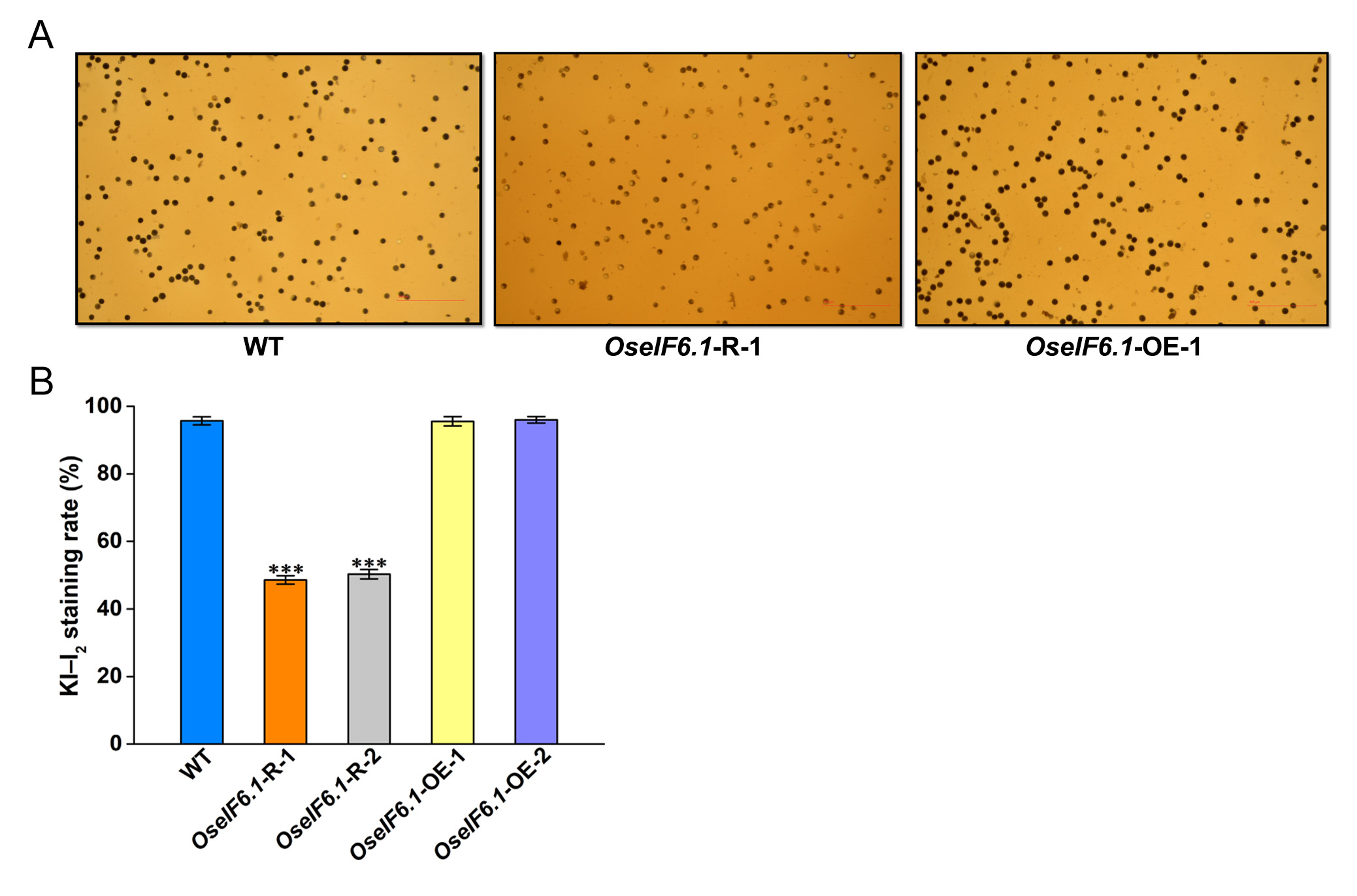

Supplement: Supplementary Figure 2 — Fertility analysis of WT and OseIF6.1 transgenic plants. (A) KI-I2 staining of WT, OseIF6.1-R-1, and OseIF6.1-OE-1 pollen. Normal pollen appears dark colored. Scale bars = 500 μm. (B) KI-I2 staining rate of WT and OseIF6.1 transgenic plants pollen. The values represent means ± SE derived from at least three independent experiments. Student’s t-test: *p < 0.05, **p < 0.01, ***p < 0.001. [file Image_2.jpeg]

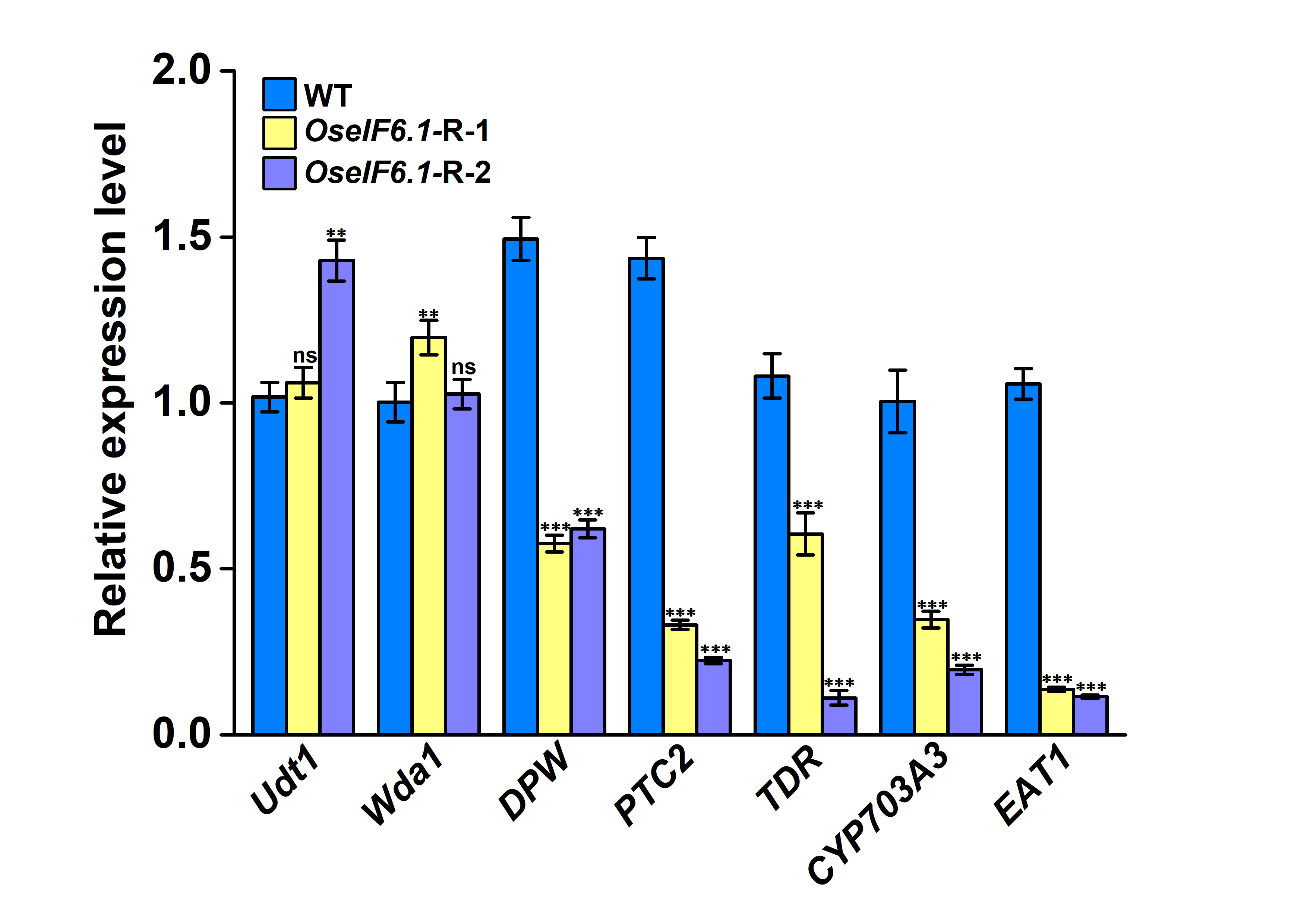

Supplement: Supplementary Figure 3 — Expression levels of genes involved in anther development in WT and OseIF6.1 knockdown transgenic plants young panicles. The values represent means ± SE derived from at least three independent experiments. Student’s t-test: *p < 0.05, **p < 0.01, ***p < 0.001. [file Image_3.jpeg]

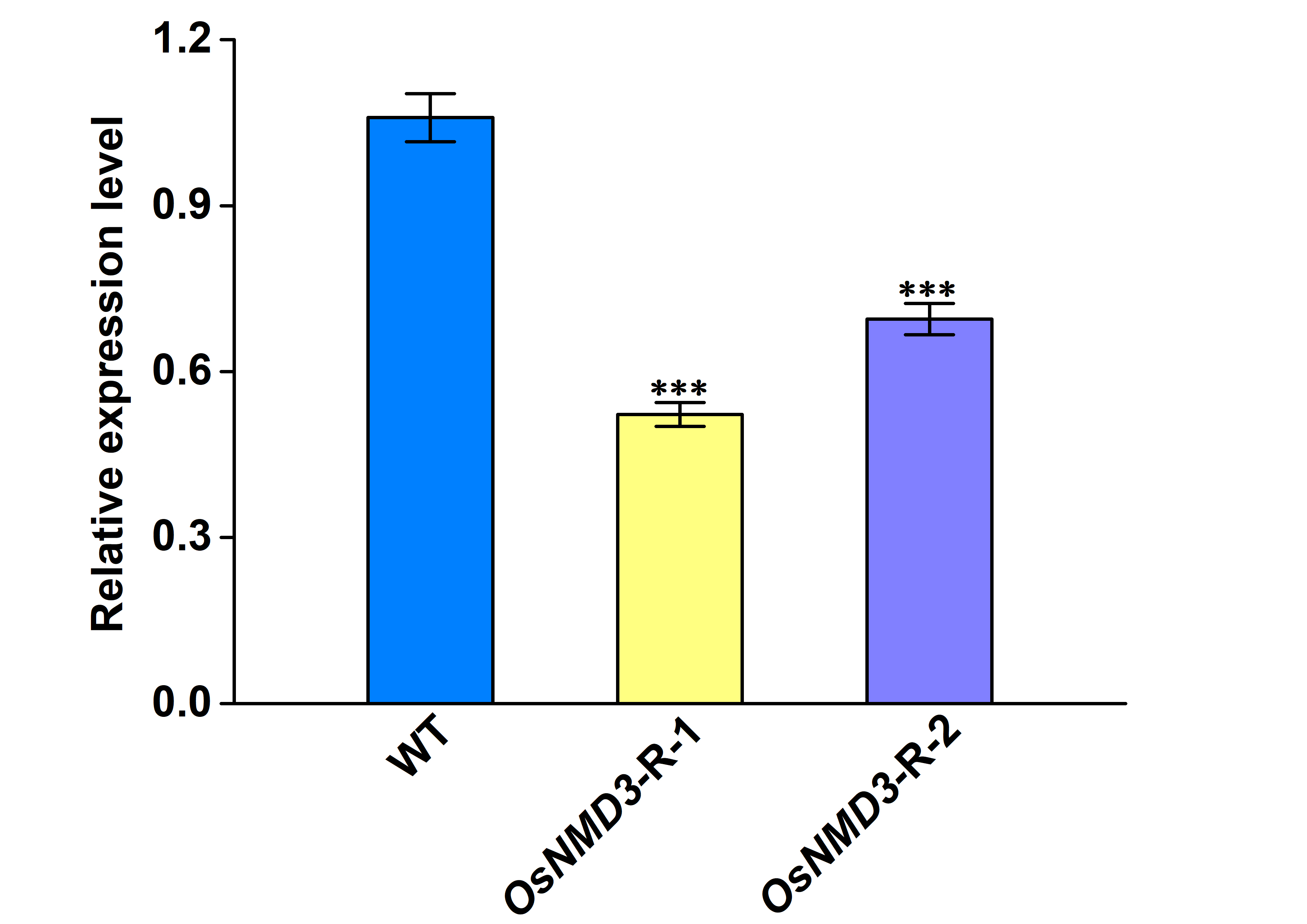

Supplement: Supplementary Figure 4 — OsNMD3 expression in WT and OsNMD3 knockdown transgenic lines. The values represent means ± SE derived from at least three independent experiments. Student’s t-test: *p < 0.05, **p < 0.01, ***p < 0.001. [file Image_4.jpeg]

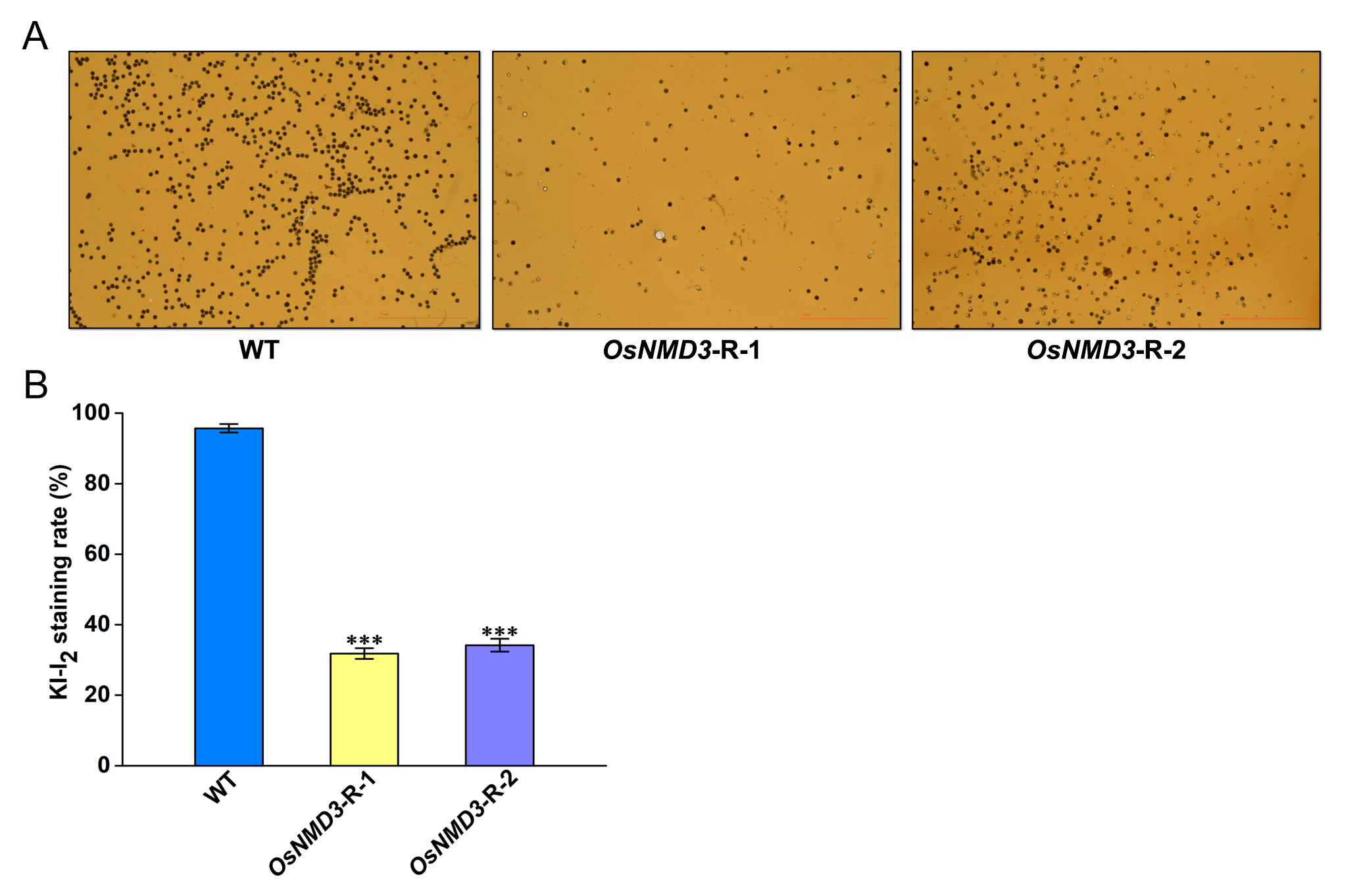

Supplement: Supplementary Figure 5 — Fertility analysis of WT and OsNMD3 knockdown transgenic plants. (A) KI-I2 staining of WT and OsNMD3 knockdown transgenic lines pollen. Normal pollen appears dark colored. Scale bars = 1 mm. (B) KI-I2 staining rate of WT and OsNMD3 knockdown transgenic lines pollen. The values represent means ± SE derived from at least three independent experiments. Student’s t-test: *p < 0.05, **p < 0.01, ***p < 0.001. [file Image_5.jpeg]

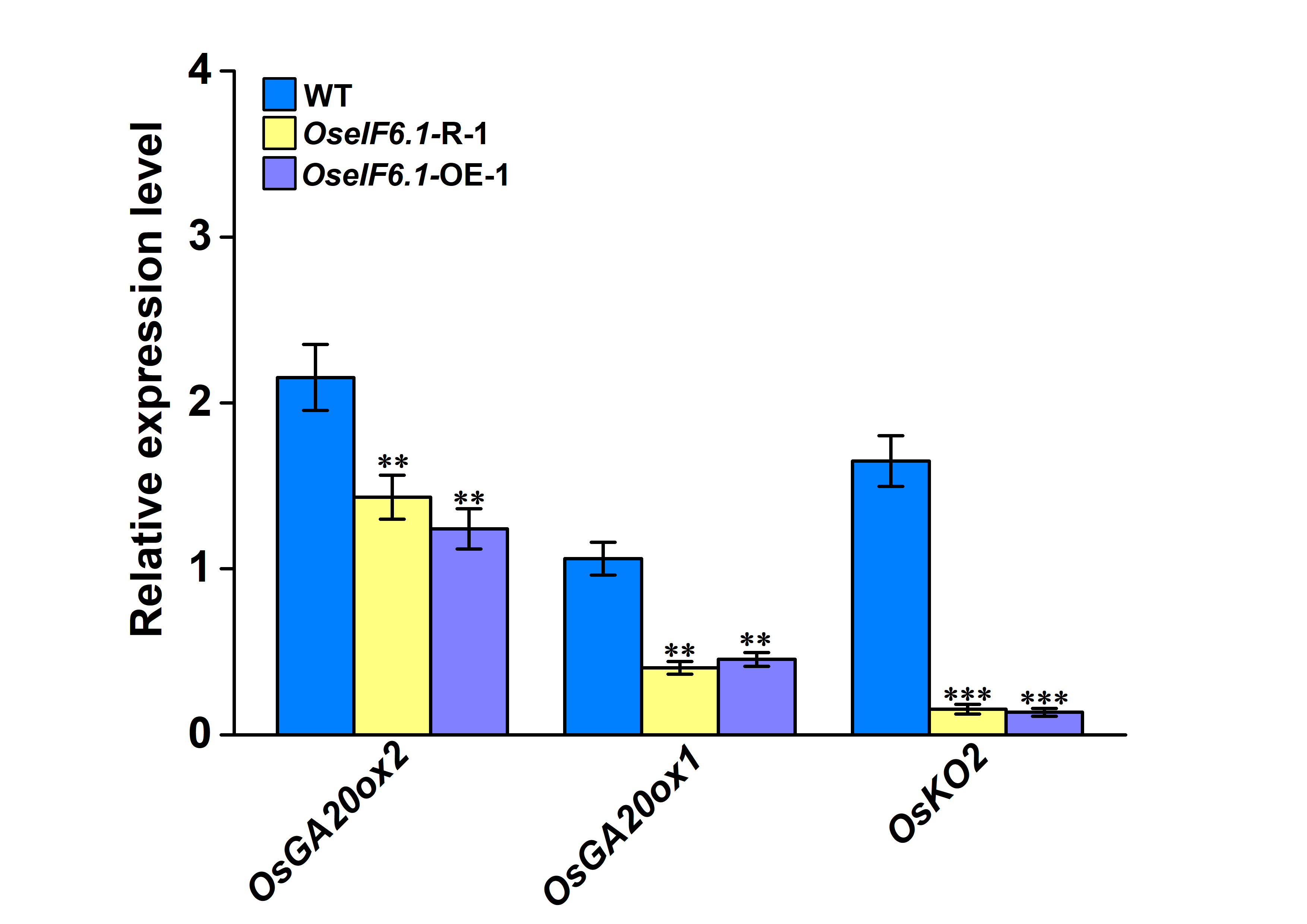

Supplement: Supplementary Figure 6 — Expression levels of genes involved in gibberellin biosynthesis in stems of WT and OseIF6.1 transgenic plants. The values represent means ± SE derived from at least three independent experiments. Student’s t-test: *p < 0.05, **p < 0.01, ***p < 0.001. [file Image_6.jpeg]

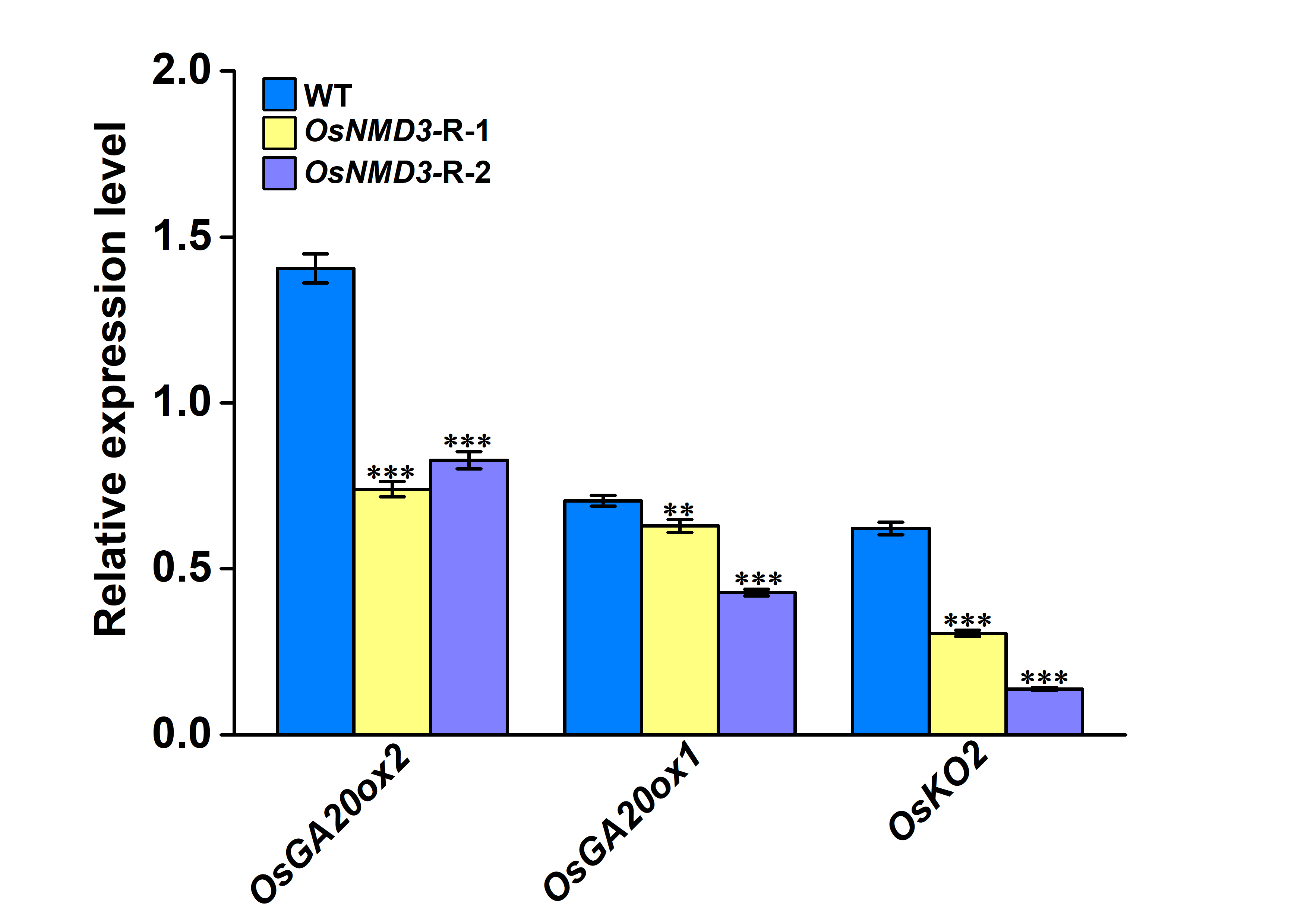

Supplement: Supplementary Figure 7 — Expression levels of genes involved in gibberellin biosynthesis in stems of WT and OsNMD3 knockdown transgenic plants. The values represent means ± SE derived from at least three independent experiments. Student’s t-test: *p < 0.05, **p < 0.01, ***p < 0.001. [file Image_7.jpeg]

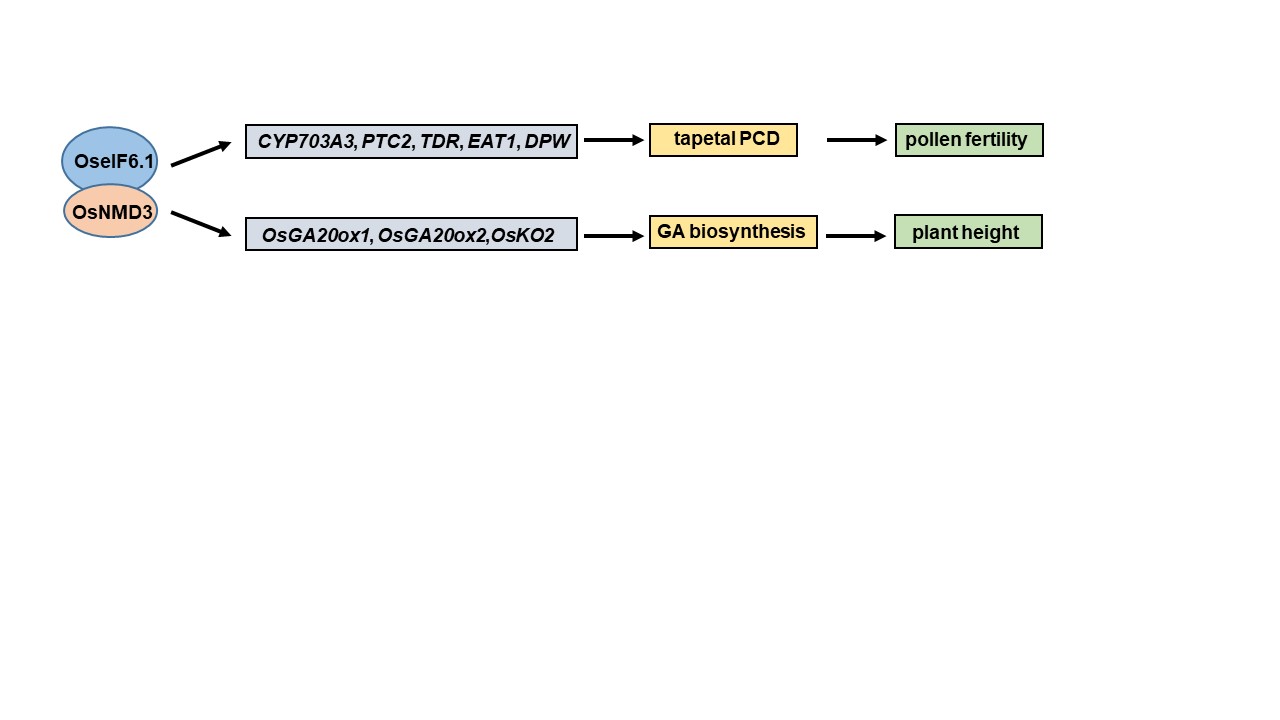

Supplement: Supplementary Figure 8 — A working model for the role of OseIF6.1 in rice growth and development. [file Image_8.jpeg]
